# ImproveAssembly - Tool for identifying new gene products and improving genome assembly

**DOI:** 10.1371/journal.pone.0206000

**Published:** 2018-10-26

**Authors:** Adonney Allan de Oliveira Veras, Bruno Merlin, Pablo Henrique Caracciolo Gomes de Sá

**Affiliations:** 1 Faculty of Computer Engineering, Federal University of Pará campus Tucuruí (CAMTUC-UFPA), Pará, Brazil; 2 Federal Rural University of Amazonia campus Tomé-Açu (UFRA), Pará, Brazil; University of Western Sydney, AUSTRALIA

## Abstract

The availability of biological information in public databases has increased exponentially. To ensure the accuracy of this information, researchers have adopted several methods and refinements to avoid the dissemination of incorrect information; for example, several automated tools are available for annotation processes. However, manual curation ensures and enriches biological information. Additionally, the genomic finishing process is complex, resulting in increased deposition of drafts genomes. This introduces bias in other omics analyses because incomplete genomic content is used. This is also observed for complete genomes. For example, genomes generated by reference assembly may not include new products in the new sequence or errors or bias can occur during the assembly process. Thus, we developed ImproveAssembly, a tool capable of identifying new products missing from genomic sequences, which can be used for complete and draft genomes. The identified products can improve the annotation of complete genomes and drafts while significantly reducing the bias when the information is used in other omics analyses.

## Introduction

Next generation sequencing platforms have greatly increased the number of genomic studies by reducing sequencing costs and thus have greatly increased the number of genomes available in public databases [1].

Compared to the Sanger sequencing method [2], NGS platforms can generate a larger amount of data, resulting in higher sequencing coverage obtained in less time [3]. The high throughput achieved by NGS platforms encouraged the development of new algorithms and programs for handling larger volumes of data and assembling genomes in a shorter time [3].

Several programs are available for performing genome assembly, such as, SPAdes [4], SOAPdenovo [5], Ray [6], Velvet [7], and ABySS [8], among others.

Many of these programs can assemble data from different sequencing platforms, but all programs show a limited ability to generate complete genomes, which remains a complex and costly task [9].

A complete genome is generated in the final step of assembly when scaffolds or contigs are combined into a complete sequence without gap regions to represent the total genomic content of the organism [10]. In contrast, a draft genome is composed of only a set of contigs or scaffolds [11].

Although they are useful for several studies, draft genomes may limit comparative genomics and structural genomics analyses because the gene content is only partially represented. Additionally, some genes may not be identified if they are in a region with poor coverage or because of assembly errors [12].

The genome finishing process is an important step in reducing information loss and leads to a more accurate representation of the genomic characteristics of the targeted organism. Determining the complete genomic sequence (genome) of microorganisms is the basis for understanding its biology and functional characterization. The complete genome can be used for analyses such as gene prediction, comparative genomics, and genome annotation [3].

Having complete or nearly complete genomes is necessary for many studies to enable more thorough genomic analyses. For example, the reliability of analyses such as operon structure identification, gene regulation, and comparative genomic studies is enhanced by the availability of complete genomes. Additionally, the finishing process can substantially improve the quality of data available to the community by identifying and correcting incorrect assemblies and low coverage regions [13,14].

Currently, several features of sequencing data, particularly increased depth of coverage and error reduction in sequencing libraries, are useful for genome finishing steps. Thus, draft genomes can be combined with additional information from new sequencing and mapping studies to reduce the effort of the finalization process [13].

The ability to understand the function of a gene and how variations affect that function depends on the understanding of the gene structure, which can be determined by genome annotation. Genome annotation is performed by automatic gene prediction algorithms, which search for gene structure patterns in a genome [15].

Numerous studies have developed methods for automated gene prediction and produced several effective algorithms for gene identification in genomes generated using *de novo* approaches. In general, these methods predict genes by learning species-specific characteristics from manually cured (manually annotated) training gene pools. These characteristics are then used to identify new genes in new assembled genomes [16].

Automated annotation programs are essential for providing an overview of the genetic content, particularly for non-model organisms, as they allow the gene content to be quickly predicted from the new genome assembly, although the results are imprecise in some cases. However, manual annotation is still considered the 'gold standard' for accurate annotation [15].

In this study we developed the computational tool ImproveAssembly, which is capable of automatically identifying gene products in complete prokaryotic genomes and drafts using raw data (reads) to improve the identification process. For draft genomes, it is possible to identify products not represented in the assembly, whereas for complete genomes ImproveAssembly identifies new gene products that are not present in the genomic sequence, increasing the accuracy of assembly and allowing for the addition of products previously not identified.

## Materials and methods

### Mapping

Bowtie v.2.3.4.1 software was used to map the raw reads against the input file. The result of this process is a file in FASTQ format containing unmapped reads. The respective parameters values used were: minins = 0, maxins = 500, mismatches in seed alignment (-N) = 0, and length of seed substrings (-L) = 22 [17].

### *De novo* assembly

Spades v.3.11.1 assembler was used to assemble the unmapped reads with default parameter values [4].

### Annotation

The Web-RAST platform was used to standardize the annotation of files. The submission, status management of the processes, and downloading of files in EMBL format was conducted through the batch interface of RAST [18].

### CDS extraction and BLAST

Extraction of coding DNA sequences (CDSs) from the EMBL files was performed using an ImproveAssembly module. The CDSs from the input file were used to construct a local Blast database.

New products were identified by a local Blast of the CDS extracted from the EMBL file based on the input file against the CDS of the EMBL file with annotated contigs produced in the assembly.

### Programming language and database

ImproveAssembly was developed in JAVA language (http://www.oracle.com/) and the Swing library was used to create the graphical interface (http://www.oracle.com/). Project management was performed with SQLite version 3 (https://www.sqlite.org/).

### Input data and workflow

ImproveAssembly can be used with both complete and draft genomes. To process complete genomes, the software requires the following input files: the genome sequence in fasta format and file with reads in the fastq format. To process genomes in the draft, the software requires the following input file: assembled contigs in format .fasta and the reads in format fastq.

**Fig 1:**
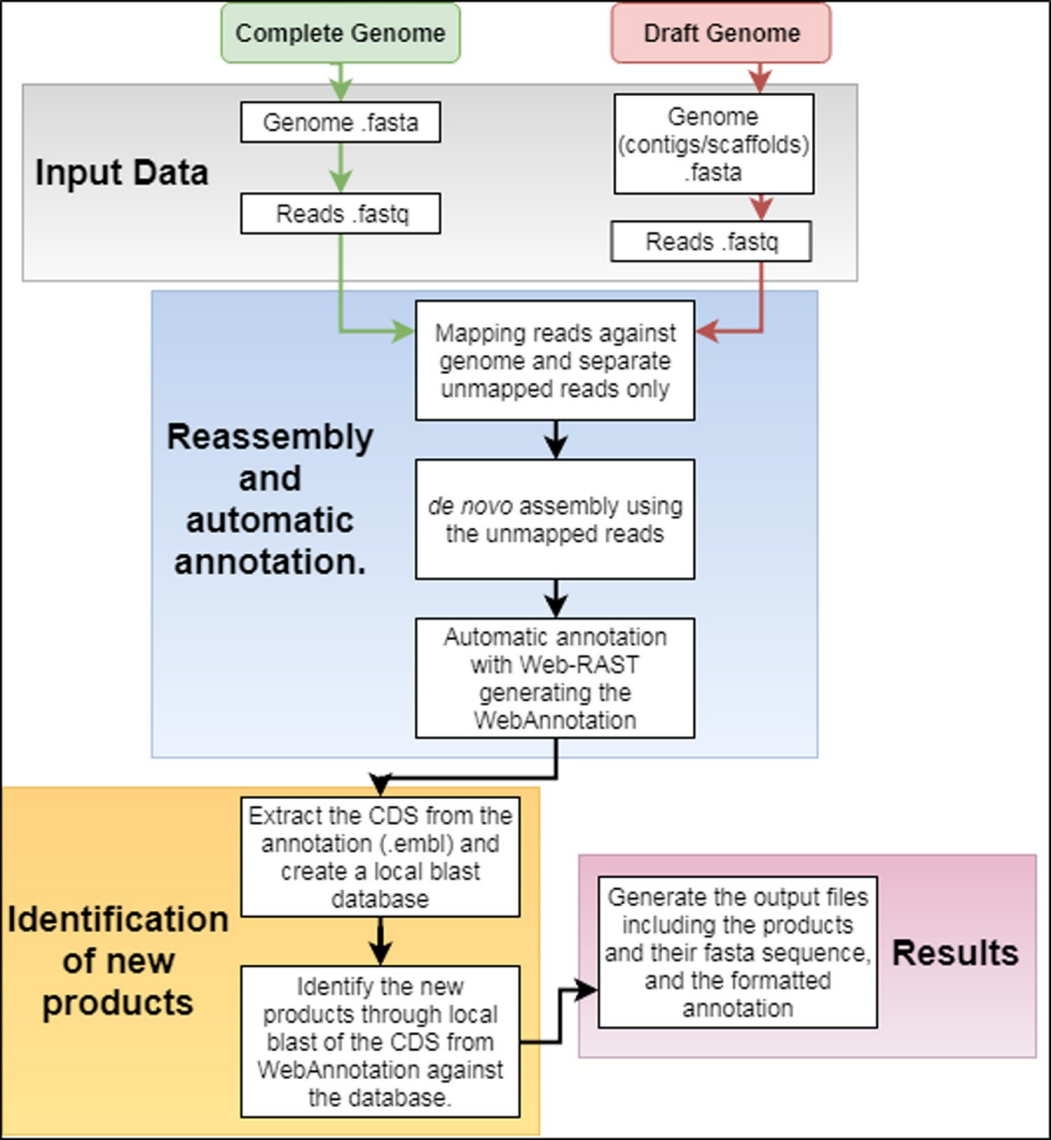
ImproveAssembly workflow. Complete genomes (green arrows), draft genomes (red arrows) and both (black arrows).

Fig 1 shows the major steps in ImproveAssembly; the green arrows indicate the path for complete genome analysis, red arrows indicate the path for draft genome analysis, and black arrows indicate the common modules between complete genomes and drafts.

The ImproveAssembly pipeline consists of the following steps: (i) Input data—Input files are: genome sequence in fasta format and reads in fastq format or genome scaffolds (contigs) in fasta format and reads in fastq format for complete genomes and draft genomes respectively. (ii) Reassembly and automatic annotation—at this step Bowtie2 is used to map the reads against the input genome file generating a file in fastq format containing the unmapped reads, then these reads are assembled using SPAdes v.3.11.1. The assembly result and genome fasta file are sent for automatic annotation on the RAST platform using the batch interface, the result of this process are two files in the EMBL format, one EMBL of the genome and one EMBL of the assembly. (iii) Identification of new products—After the EMBL files are downloaded, the CDS from the genome EMBL are extracted and used in the construction of the local database, BLAST is used in the mapping of the CDS extracted from the assembly EMBL against the local database, products that did not get hits on BLAST analysis represent the new products identified. (iv) Results—three files are generated, a file in fasta format containing the new products, a tabular file containing the locus_tag of the product and the function predicted through RAST, and a report in pdf format.

### Tool validation

To validate ImproveAssembly were used data from thirteen organisms. Four strains of *Mycobacterium tuberculosis*, two *Kineococcus* and seven strains of *Escherichia* coli. Each organism with its SRA number is listed in Table 1. All organisms had their genome downloaded directly from the NCBI, with the exception of the *Escherichia coli* 042 that had its genome assembled by SPADES from the raw reads available in the SRA.

**Table 1:** Organisms and SRA number used to validate ImproveAssembly.

Organism	SRA Acess Number
*Mycobacterium tuberculosis* F11	SRR974839
*Mycobacterium tuberculosis* KZN 1435	SRR1144793
*Mycobacterium tuberculosis* str. Haarlem	SRR974846
*Mycobacterium tuberculosis* KZN 605	SRR857301
*Kineococcus rhizosphaerae* DSM 19711	SRR6479489
*Kineococcus xinjiangensis* DSM 22857	SRR6479482
*Escherichia coli* RR1	SRR2014554
*Escherichia coli* KLY	SRR1424625
*Escherichia coli* P12b	SRR2000272
*Escherichia coli* K-12	SRR2537294
*Escherichia coli* O157:H7	SRR3223744
*Escherichia coli* 'BL21-Gold(DE3)pLysS AG'	SRR4240341
*Escherichia coli* 042	ERR007646

The *E*. *coli RR1*, *E*. *coli KLY*, *E*. *coli P12b* and *E*. *coli K-12*, despite being complete genomes, had their reads assembled by SPADES, in order to be submitted to ImproveAssembly as draft genome. The results of the assembly are shown in Table 2.

**Table 2:** Results of the assembly process with SPADES.

Organism	N50	Larger Contig	Smaller Contig	Contigs	Total of bases
*Escherichia coli RR1*	140.760	409.569	181	144	4.529.368
*Escherichia coli KLY*	148.396	326.983	81	98	4.644.355
*Escherichia coli P12b*	94.587	244.438	184	180	4.757.688
*Escherichia coli K-12*	132.490	347.926	220	170	4.578.480

### Comparative analysis

To evaluate the application of ImproveAssembly, two comparative genomic analyses were performed using the PanWeb tool [19]. The input of these analyses were as follows. For the first analysis, a file in EMBL format of the seven strains of *e*. *coli* without products identified by ImproveAssembly was used. For the second analysis, for the EMBL of the same seven strains with new products identified by ImproveAssembly, both files were previously annotated on the RAST platform.

## Results

ImproveAssembly generates three files: a file in FASTA format containing the new products, tabular file containing the locus_tag of the product and function predicted through RAST (Table 3), and report in .pdf format showing the products identified, quantity of products with function, and hypothetical proteins. These files can be used in articles or other publications.

**Table 3:** Example of *E*. *coli RR1* tabular file containing the locus_tag and products identified by ImproveAssembly.

Locus_Tag	Function predicted with Rast
SRR2014554_complete_assembly_1590	FIG00640785: hypothetical protein
SRR2014554_complete_assembly_2482	hypothetical protein
SRR2014554_complete_assembly_1492	hypothetical protein
SRR2014554_complete_assembly_0217	hypothetical protein
SRR2014554_complete_assembly_0701	FIG00640293: hypothetical protein
SRR2014554_complete_assembly_1789	hypothetical protein
SRR2014554_complete_assembly_0308	hypothetical protein
SRR2014554_complete_assembly_0604	hypothetical protein
SRR2014554_complete_assembly_0573	FIG01045439: hypothetical protein
SRR2014554_complete_assembly_3104	Gene D protein
SRR2014554_complete_assembly_1077	hypothetical protein
SRR2014554_complete_assembly_3348	hypothetical protein
SRR2014554_complete_assembly_0043	hypothetical protein
SRR2014554_complete_assembly_1275	hypothetical protein
SRR2014554_complete_assembly_3613	hypothetical protein
SRR2014554_complete_assembly_4327	hypothetical protein
SRR2014554_complete_assembly_2682	hypothetical protein
SRR2014554_complete_assembly_0962	Hypothetical response regulatory protein ygeK
SRR2014554_complete_assembly_2451	hypothetical protein
SRR2014554_complete_assembly_4223	FIG00641106: hypothetical protein
SRR2014554_complete_assembly_2991	C4-dicarboxylate transporter DcuC (TC 2.A.61.1.1)
SRR2014554_complete_assembly_3322	Mobile element protein
SRR2014554_complete_assembly_4169	Ferredoxin

The FASTA file with new products, generated by ImproveAssembly, can be used immediately for further bioinformatics analysis or as inputs in other programs.

The program was found to be efficient in identifying new products for all organisms present in the analysis. The results do not depend on whether the genome is completed or a draft, as shown in Table 4.

**Table 4:** Quantity of new products for all thirteen organisms. Total amount of products in the input genome. Total of new products identified for each organism, along with quantity of products with function already described, amount of hypothetical proteins and genome status.

Organism	Total products in the input Genome	Total identified new products	Products with function	Hypothetical protein	Genome Status
*E*. *coli RR1*	4323	23	5	18	Complete
*E*. *coli KLY*	4306	21	1	20	Complete
*E*. *coli P12b*	4298	47	12	35	Complete
*E*. *coli K-12*	4306	17	6	11	Complete
*E*. *coli O157*:*H7*	5317	98	36	62	Complete
*E*. *coli* *'BL21-Gold(DE3)pLysS AG'*	4154	10	5	5	Complete
*E*. *coli 042*	4369	38	16	22	Draft
*Mycobacterium tuberculosis F11*	4501	33	2	31	Complete
*Mycobacterium tuberculosis KZN 1435*	4920	28	2	26	Complete
*Mycobacterium tuberculosis str*. *Haarlem*	4353	169	98	71	Complete
*Mycobacterium tuberculosis KZN 4207*	5756	23	2	21	Complete
*Mycobacterium tuberculosis KZN 605*	4374	4	1	3	Complete
*Kineococcus rhizosphaerae DSM 19711*	5157	31	9	22	Draft
*Kineococcus xinjiangensis DSM 22857*	4323	18	8	10	Draft

Among the results, the organism showing the lowest number of new products with functions was *Escherichia coli KLY* and *Mycobacterium tuberculosis KZN 605*, for which only one product was identified, respectively, while the other 20 and 3 were hypothetical proteins. In *E*. *coli O157*:*H7*, a total of 98 new products were identified; the functions of 36 of these products have already been described, which can aid in improving assembly and annotation of this genome, while 62 proteins were hypothetical. The highest result in number of products was *Mycobacterium tuberculosis str*. *Haarlem* in which 169 products were identified, 98 with function already described and 71 hypothetical proteins.

The number of new products identified in draft genomes is expected to be greater than those in complete genomes, as complete genomes are further analyzed and finalized. However, there are exceptions, such as *E*. *coli O157*:*H7 and Mycobacterium tuberculosis str*. *Haarlem*; although its complete genome is known, numerous new products were identified.

In all organisms evaluated, the number of hypothetical proteins was greater than the number of products with function. Still, this is a promising result considering that new products with functions were identified for all organisms.

The *E*. *coli RR1*, *E*. *coli KLY*, *E*. *coli P12b* and *E*. *coli K-12*, that were assembled and used as draft for ImproveAssembly also achieved good results, as shown in Table 5, where new products were identified for all four stains. These results reinforce that the ImproveAssembly identifies new products for draft genomes and complete genomes.

**Table 5:** ImproveAssembly result for the *e*. *coli* strains used as draft. Shows the otal amount of products in the input genome, total of new products identified for each organism, along with quantity of products with function already described, amount of hypothetical proteins and the genome status.

Organism	Total products in the input Genome	Total identified new products	Products with function	Hypothetical protein	Genome Status
*E*. *coli RR1*	4339	11	3	8	Draft
*E*. *coli KLY*	4468	13	4	9	Draft
*E*. *coli P12b*	4676	21	5	16	Draft
*E*. *coli K-12*	4353	13	2	11	Draft

**Fig 2:**
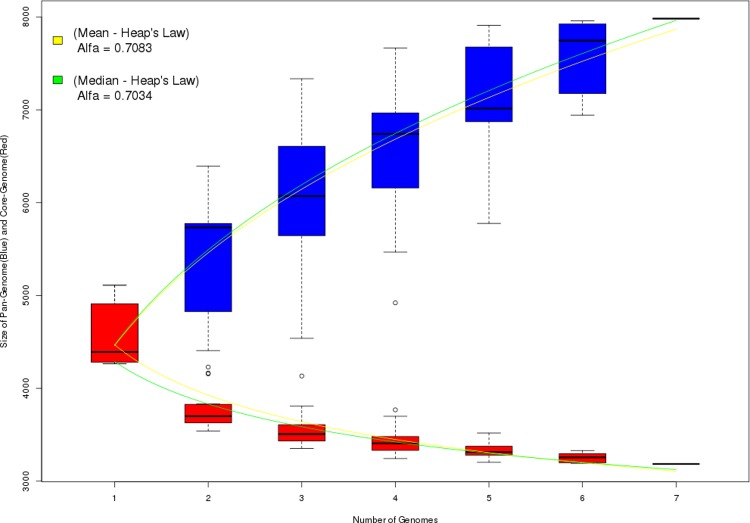
Pangenomic analysis of seven *E*. *coli* strains without the presence of new products identified by ImproveAssembly.

**Fig 3:**
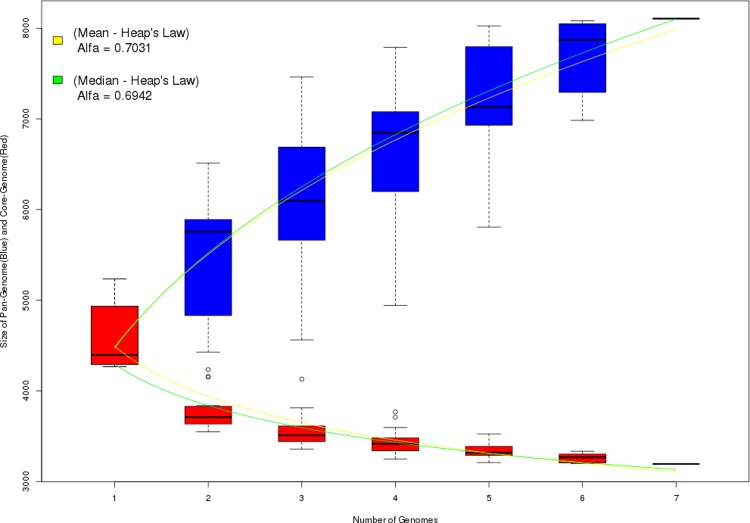
Pangenomic analysis of seven *E*. *coli* strains with the addition of new products identified by ImproveAssembly.

In comparative analysis, Figs 2 and 3 shows the results of pangenomic analysis of the seven strains of *E*. *coli*. Fig 2 shows the results of analyzing the strains without modification, that is, without the new products identified by the ImproveAssembly pipeline. Fig 3 shows the results after adding the new products identified in each strain by ImprovedAssembly.

According to Heap's Law, the pangenome is considered open if the value of alpha (α) is less than or equal to one (α ≤ 1) and closed to alpha values greater than one (α > 1) [20]. Comparison of the alpha values of the mean and median of the results revealed values close to zero (Fig 3).

Thus, addition of the new products identified by ImproveAssembly and added to the analysis greatly increased the number of genes even without adding new genomes. This was confirmed by the alpha values calculated for both analyses. These results demonstrate the efficiency of the pipeline in identifying new products and the impact of this method on other omics analyses.

## Discussion

The ImproveAssembly program was effective for enhancing genome assembly and identifying missing products by identifying new products in both complete and draft genomes. These types of tools are necessary to generate more complete genomes with more accurate information, which will directly impact analyses such as comparative genomics, gene expression, and phylogenomics, among others.

For comparative genomics, we demonstrated that new products identified by ImproveAssembly positively influenced the final analysis results, generating better results compared to analysis conducted without the new ImproveAssembly products.

The great advantage of ImproveAssembly is that it is fully automated. The user is only required to insert the assembly data or contigs and reads. The graphical interface of the program is user-friendly and easy to use even for those with no background in computing. The program also controls the steps involving databases and can resume the process in case of an external error. Furthermore, ImproveAssembly uses RAST annotation, which is a widely used and established genome annotation web server in the bioinformatics community.

Additionally, the software presents two execution modes. The first allows for analysis of only one genome at a time. The second one allows for multiple genomes to be added with their respective reads, and the pipeline evaluates all organisms sequentially, which is ideal for overnight analysis. It is important to note that ImproveAssembly is currently only available for prokaryotes.
